# Proton spectroscopy: a simple and useful tool in the investigation of mitochondrial disease

**DOI:** 10.1590/0004-282X-ANP-2021-0422

**Published:** 2022-04-22

**Authors:** Daniel Venturino NASSIF, Luiz Felipe Rocha VASCONCELLOS

**Affiliations:** 1 Hospital Federal dos Servidores do Estado, Serviço de Neurologia, Rio de Janeiro, RJ, Brazil.

A 38-year-old woman presented to the emergency department with right hemiparesis. Brain computed tomography (CT) and magnetic resonance imaging (MRI) were recommended (Figures 1 and 2). Genetic study confirmed the presence of a point mutation m.3243 A>G of mtDNA, confirming the diagnosis of Mitochondrial myopathy, Encephalopathy, Lactic Acidosis, and Stroke-like episodes (MELAS).

**Figure 1.**
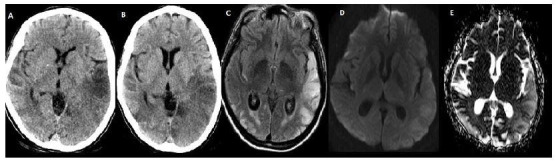
Brain computed tomography during hospital admission (A), 72 hours later (B), and fluid attenuated inversion recovery imaging (C) demonstrating bilateral lesions that do not respect arterial vascular territories. Diffusion-weighted imaging and apparent diffusion coefficient (D–E) demonstrating T2 shine through effect, representing vasogenic rather than cytotoxic edema.

**Figure 2.**
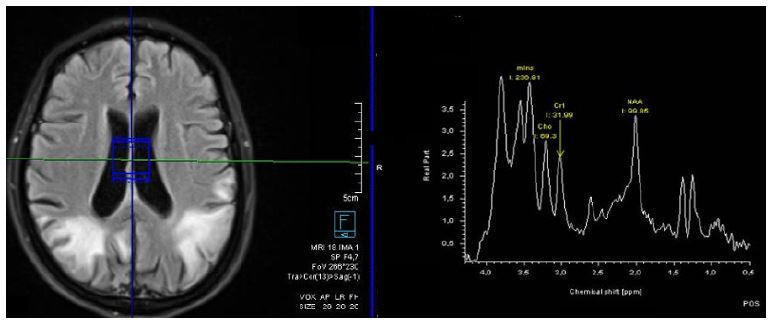
Magnetic resonance imaging spectroscopy demonstrates elevated lactate peak in cerebrospinal fluid.

MELAS is a rare mitochondrial disorder that can be manifested by stroke-like episodes, epilepsy, hyperlactatemia, myopathy, headaches, deafness, diabetes, and short stature^1^. Although not pathognomonic, the lactate peak observed in spectroscopy can be an indication for MELAS diagnosis, which is found to be correlated with the severity of clinical manifestations^2^. Genetic testing confirms the diagnosis^3^.

## References

[1] El-Hattab AW, Adesina AM, Jones J, Scaglia F (2015). MELAS syndrome: Clinical manifestations, pathogenesis, and treatment options. Mol Genet Metab..

[2] Kaufmann P, Shungu DC, Sano MC, Jhung S, Engelstad K, Mitsis E (2004). Cerebral lactic acidosis correlates with neurological impairment in MELAS. Neurology..

[3] Lorenzoni PJ, Werneck LC, Kay CSK, Silvado CES, Scola RH (2015). When should MELAS (Mitochondrial myopathy, Encephalopathy, Lactic Acidosis, and Strokelike episodes) be the diagnosis?. Arq Neuro-Psiquiatr..

